# Short‐Interval Discordance Between Fatigue and Joint Counts in Rheumatoid Arthritis

**DOI:** 10.1111/1756-185x.70887

**Published:** 2026-09-29

**Authors:** Yidi Huang, Lü Liu, Hangwei Wan, Cunxiang Xie, Hailong Wang

**Affiliations:** ^1^ Department of Rheumatology, Dongzhimen Hospital Beijing University of Chinese Medicine Beijing China; ^2^ Dongzhimen Hospital Beijing University of Chinese Medicine Beijing China; ^3^ Aerospace Central Hospital Beijing China

Dear Editor,

Fatigue is a core patient‐reported outcome in rheumatoid arthritis (RA), but it is only partly explained by inflammatory markers and joint examination [1, 2, 3]. Most longitudinal studies have assessed fatigue over several weeks or months [4, 5]. We examined change in fatigue visual analogue scale (VAS) at the second routine assessment, which often occurred within days, and evaluated whether a decrease in fatigue occurred in parallel with changes in swollen joint count (SJC) and tender joint count (TJC).

We conducted a retrospective single‐centre study of hospitalized patients with RA using a de‐identified routine‐care database from the Department of Rheumatology, Dongzhimen Hospital, Beijing University of Chinese Medicine. Records included in the analysis spanned 12 May 2022 to 18 December 2024. Eligible inpatient records were identified consecutively, and all records meeting the study‐specific criteria were included without sampling. Patients were eligible if RA was diagnosed according to the 2010 ACR/EULAR criteria or confirmed by a rheumatologist and fatigue VAS was recorded at baseline and at the second assessment [6]. Baseline was the assessment used for cohort entry, and the second assessment was the next recorded routine‐care assessment after baseline. Each patient contributed one assessment pair. Reporting followed STROBE guidance [7]. Fatigue was recorded on a 0–10 VAS, with 0 indicating no fatigue and 10 extreme fatigue. SJC and TJC were obtained from routine 28‐joint examinations covering the bilateral shoulders, elbows, wrists, metacarpophalangeal joints, proximal interphalangeal joints and knees. A fatigue decrease was defined as a reduction of at least 1 point on the 0–10 fatigue VAS, consistent with published estimates of minimally important improvement in RA [8]. Because a ≥ 1‐point decrease was not possible from a baseline fatigue VAS of 0, the primary fatigue‐responder analysis was restricted to patients with baseline fatigue VAS ≥ 1 (*n* = 189). The 30 patients with baseline fatigue VAS = 0 were retained in the full‐cohort analyses and classified as having no ≥ 1‐point decrease. The four‐pattern fatigue–joint count classification used the full cohort (*N* = 219). The primary joint‐count comparator was any decrease in SJC or TJC. Sensitivity analyses required simultaneous SJC and TJC decrease, at least 20% reduction in either count, or at least 20% reduction in both counts. Continuous paired outcomes were summarized at both assessments and as within‐patient change (second assessment minus baseline) and were compared using the Wilcoxon signed‐rank test. Paired CRP and ESR analyses used complete paired observations only; missing laboratory values were not imputed. Binary proportions were reported with Wilson 95% confidence intervals. Exploratory logistic regression models were fitted separately for each candidate predictor. Models for disease‐activity measures were adjusted for age, sex, body mass index (BMI) and baseline fatigue VAS. In the BMI model, BMI was the predictor of interest and age and sex were the only covariates. These regression analyses were exploratory and were not adjusted for multiple comparisons. DAS28, CDAI and SDAI were treated as established RA disease‐activity indices [9, 10, 11].

The analysis included 219 patients; 156 (71.2%) were women, and the mean age was 61.87 years (SD 11.90). Second‐assessment dates were available for 187 patients. Two records with chronologically inconsistent or negative intervals were excluded from the interval summary, leaving 185 patients with a median interval of 7 days (IQR 6–9). All 219 patients had paired fatigue VAS, SJC and TJC measurements and contributed to the paired fatigue–joint count analyses; the interval summary was therefore restricted to 185 patients.

Fatigue VAS decreased from a median of 3.00 (IQR 1.50–5.00) to 2.00 (IQR 1.00–4.00; *p* < 0.001). CRP also decreased among the 163 patients with paired CRP measurements (Table 1), whereas ESR in 144 paired observations did not show a statistically significant group‐level change. SJC and TJC likewise showed no statistically significant group‐level change, although 119 of 219 patients (54.3%, 95% CI 47.7–60.8) had a decrease in at least one joint count at the individual level. Among the 189 patients with baseline fatigue VAS ≥ 1, 166 achieved a ≥ 1‐point fatigue decrease (87.8%, 95% CI 82.4–91.8), which was the primary fatigue‐responder estimate. In the full cohort, the corresponding descriptive estimate was 166/219 (75.8%, 95% CI 69.7–81.0).

**TABLE 1:** Short‐interval changes and principal fatigue outcomes.

Panel A: paired continuous measures				
*n*	Baseline, median (IQR)	Second assessment, median (IQR)	Within‐patient change, median (IQR)	*p*
219	3.00 (1.50–5.00)	2.00 (1.00–4.00)	−1.00 (−1.00 to −1.00)	< 0.001
163	13.03 (4.03–37.98)	6.13 (1.40–19.15)	−5.99 (−23.32 to 5.55)	< 0.001
144	36.00 (21.00–62.25)	30.00 (17.00–58.00)	−6.00 (−30.25 to 16.00)	0.084
219	10.00 (4.00–16.00)	10.00 (4.00–16.00)	0.00 (−6.50 to 7.00)	0.954
219	13.00 (8.00–18.00)	12.00 (8.00–18.00)	0.00 (−7.00 to 7.00)	0.868

Panel B: principal binary fatigue outcomes			
Outcome	Denominator	*n* (%)	95% CI
≥ 1‐point fatigue decrease, baseline fatigue VAS ≥ 1 (primary)	189	166 (87.8%)	82.4–91.8
≥ 1‐point fatigue decrease, full cohort (descriptive)	219	166 (75.8%)	69.7–81.0
Fatigue decrease without any SJC or TJC decrease	219	77 (35.2%)	29.1–41.7

*Note:* The reassessment interval was available for 185 patients and had a median of 7 days (IQR 6–9). Within‐patient change was calculated as second assessment minus baseline. CRP and ESR analyses included complete paired observations only; missing values were not imputed. *p* Values for paired continuous outcomes were obtained using the Wilcoxon signed‐rank test. Proportion 95% CIs were calculated using the Wilson method.

Abbreviations: CI, confidence interval; CRP, C‐reactive protein; ESR, erythrocyte sedimentation rate; IQR, interquartile range; SJC, swollen joint count; TJC, tender joint count; VAS, visual analogue scale.

The four fatigue‐joint count patterns are shown in Figure 1. Fatigue decreased together with any joint‐count decrease in 89 patients (40.6%, 95% CI 34.3–47.3), whereas 77 (35.2%, 95% CI 29.1–41.7) had a fatigue decrease without a decrease in either SJC or TJC. Thirty patients (13.7%, 95% CI 9.8–18.9) had a joint‐count decrease without a fatigue decrease, and 23 (10.5%, 95% CI 7.1–15.3) had neither. Under the alternative joint‐count definitions, the fatigue‐only pattern occurred in 105/219 patients (47.9%, 95% CI 41.4–54.5) when both SJC and TJC were required to decrease, 85/219 (38.8%, 95% CI 32.6–45.4) when a ≥ 20% reduction in either count was required, and 115/219 (52.5%, 95% CI 45.9–59.0) when a ≥ 20% reduction in both counts was required.

**FIGURE 1:**
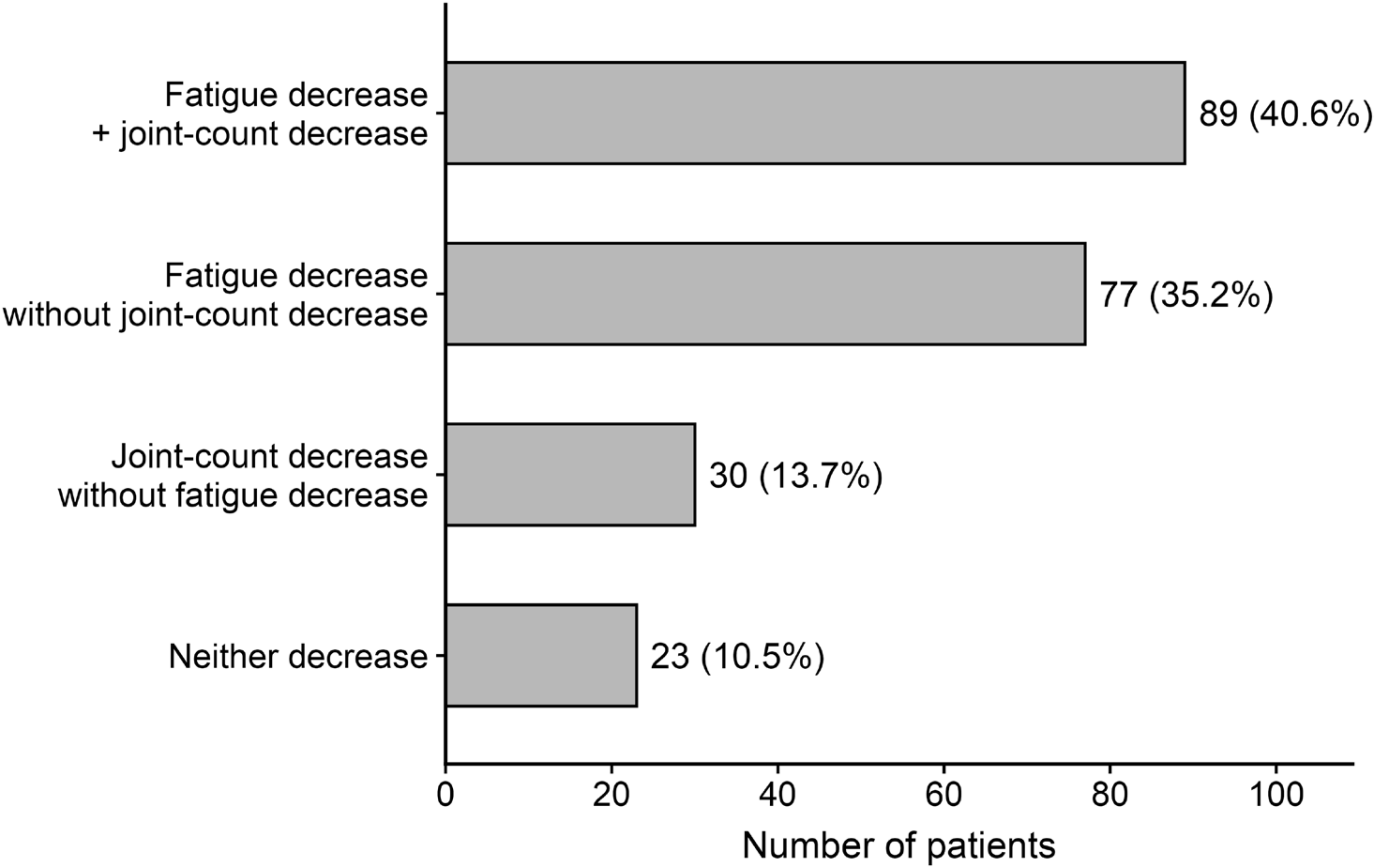
Classification of ≥ 1‐point fatigue decrease and any joint‐count decrease in the full cohort (*N* = 219). A fatigue decrease was defined as a decrease of at least 1 point on the fatigue VAS. Any joint‐count decrease was defined as a decrease in SJC or TJC. The four categories are mutually exclusive and exhaustive. This comparator was descriptive and was not intended as a validated clinical joint‐response criterion.

Higher baseline DAS28‐CRP (adjusted odds ratio [OR] 1.55, 95% CI 1.02–2.35), DAS28‐ESR (OR 1.81, 95% CI 1.14–2.88), and SJC (OR 1.09 per joint, 95% CI 1.02–1.17) were associated with failure to achieve a 1‐point fatigue decrease. Associations for CDAI, SDAI, and TJC were weaker, and ESR and C‐reactive protein alone were not associated with this outcome.

The results show partial, rather than complete, concordance between early changes in fatigue and joint counts. The absence of a statistically significant group‐level change in SJC or TJC does not imply that individual joint counts were unchanged, because decreases and increases can coexist within the cohort. This is consistent with evidence that RA fatigue reflects interacting inflammatory, physical, and psychosocial factors [2, 3, 12, 13, 14]. The finding does not indicate that fatigue is independent of RA activity: baseline composite disease activity and joint burden were associated with fatigue at the second assessment. It does indicate that a short‐term change in fatigue cannot be inferred from joint‐count change alone.

Several limitations are relevant. Valid reassessment intervals were available for 185 of 219 patients; therefore, the median 7‐day interval does not describe the entire cohort. Treatment changes during the short interval were not captured consistently, so the observed fatigue decrease cannot be attributed to a specific therapy. Rest, sleep, clinical contact, measurement variation, and regression to the mean may also have contributed. The study was conducted at one centre; fatigue was measured with a single‐item VAS, and information on sleep, mood, and physical activity was unavailable. The broad primary joint‐count definition was addressed with stricter sensitivity analyses, but single‐joint differences and routine examination variability may affect individual classifications, and none of these definitions constitutes a validated short‐term joint‐response criterion.

In routine reassessment, fatigue VAS may complement joint examination and composite disease‐activity measures. A decrease in fatigue over a few days should be interpreted as a patient‐reported change, not as evidence of inflammatory control or treatment efficacy.

## Author Contributions

Yidi Huang contributed to study conception, data curation, statistical analysis, interpretation, and drafting. Lü Liu, Hangwei Wan, and Cunxiang Xie contributed to data collection, data checking, and manuscript revision. Hailong Wang supervised the study design, quality control, interpretation, and final revision. All authors read and approved the final manuscript.

## Funding

This study was supported by the Basic Scientific Research Fund of Beijing University of Chinese Medicine (Jiebang Guashuai Project, No. 2024‐JYB‐JBZD‐023; New Faculty Startup Fund Project, No. 2026‐JYB‐XJSJJ044).

## Ethics Statement

The Ethics Committee of Dongzhimen Hospital Affiliated to Beijing University of Chinese Medicine approved the study (approval no. 2025DZMEC‐036‐02). The database was de‐identified, and the requirement for informed consent was waived by the ethics committee.

## Consent

The authors have nothing to report.

## Conflicts of Interest

The authors declare no conflicts of interest.

## Data Availability

The datasets are not publicly available because they contain potentially sensitive clinical information and are subject to institutional data‐governance restrictions. De‐identified data may be available from the corresponding author upon reasonable request and with permission from the relevant institutional authority.
